# Distribution and dynamics of mitochondrial DNA methylation in oocytes, embryos and granulosa cells

**DOI:** 10.1038/s41598-019-48422-8

**Published:** 2019-08-15

**Authors:** Marc-André Sirard

**Affiliations:** Centre de recherche en reproduction, développement et santé intergénérationnelle (CRDSI) Département des Sciences Animales, Faculté des sciences de l’agriculture et de l’alimentation, Université Laval, Québec, Canada

**Keywords:** Energy metabolism, DNA methylation

## Abstract

Comparison of mitochondrial DNA (mtDNA) methylation patterns in oocytes, blastocysts and ovarian granulosa cells indicates hitherto unsuspected dynamics. Oocytes and blastocysts recovered from cows subjected to ovarian stimulation and from non-stimulated abattoir ovaries were analyzed using bisulphite transformation of DNA followed by whole genome sequencing. The cow is a recognized as a good model for human oocyte and pre-implantation development. The number of mtDNA copies is high in oocytes (200,000–400,000) and early embryos, resulting in very high coverage (>3000x) and very low p values for each of 716 cytosine-based nucleosides. Methylation ratio was lowest in oocytes, following by blastocysts then granulosa cells and was not restricted to CG sites but was found also at CHG and CHH sites. The initial methylation pattern is conserved during the first week of life but not in somatic cells. RNA analysis of mitochondria encoded genes showed a significant inverse correlation between methylation and expression for almost all sequences. Methylation was more extensive in somatic tissues from mature animals than in immature pre-pubertal animals. Our findings suggest that mtDNA methylation might play a programming role during gametogenesis and would be subject to epigenetic regulation according to environment and/or maternal maturity.

## Introduction

Mitochondria play an important role in all animal cells as a metabolic energy provider, as well as a regulator of many cellular processes including apoptosis or cell suicide^1^. They are present in all tissues, including in oocytes where they go through a selection bottleneck to ensure the passage of the most functional organelles to the next generation^2^. It should be kept in mind that mitochondria are not transmitted via spermatozoa to the offspring^3^. The role of mitochondria in mammalian oocytes is complex and changes as the organelles acquire a unique morphology associated with a certain level of quiescence^4^. Several studies indicate that mitochondrial function is important both in peri-fertilization events in oocytes and in post-fertilization events in early embryos^5^. A peculiar feature of mitochondria is possession of a genome that encodes only a few of the thousands of genes required for their function. In the case of mammalian cells, mitochondrial DNA (mtDNA) encodes 13 protein components of the oxidative phosphorylation system (subunits of complexes I, III, IV, and V), as well as mitochondria-specific small and large ribosomal RNA fragments and an array of 22 transfer RNA molecules^6^. Continuous crosstalk between mitochondria and the nucleus is necessary to ensure that both organelles mobilize the right components for proper function. Mitochondrial genes in the nucleus are under the same type of regulation as non-mitochondrial genes, in which transcription factors, histones and the chromatin environment determine the level of expression of mRNA and ensure its transfer to the cytoplasm for translation, as well as incorporation of the resulting protein into organelles^7^. On the other hand, the structure of the mitochondrial genome is quite different, consisting of a single 16 kb circular molecule that lacks nucleosomes or histones but rather self-organizes into structures called nucleoids^8^. The genes are positioned in a polycistronic sequence, and transcription as well as replication begins in the region called the D-Loop, in which the regulatory sequences are located without exons^9^. Since mitochondrial DNA is not part of the reference system used to analyse sequencing data, these genes are rarely included in microarrays, and therefore, little is known about their expression^10^. In reproductive tissues, they are rarely studied except in association with specific mitochondrial dysfunctions or mutations in humans^11^.

A related area of study on oocyte mitochondria concerns the transmission of defective organelles in specific metabolic conditions. For example, female mice fed a high-fat diet display modified mitochondrial morphology and function^12^. Such defects can be induced by several metabolic conditions and are believed to participate in the transmission of F_1_ phenotypes associated with mitochondrial dysfunction^13^. However, it is not known if these changes are associated with the nuclear or the mitochondrial genome. It is known that metabolic disorders in many mammalian species can be transmitted through epigenetic inheritance via the oocyte or the sperm cell^14^. Although it is technically difficult to measure DNA methylation in individual oocytes^15,16^, the information obtained so far indicates that epigenetic components (histones and DNA methylation) may play a role in metabolic programming of the future individual^17^. It has been shown that babies conceived via assisted reproductive technologies may display some phenotypical alterations (glucose levels, insulin levels, blood pressure), however, it is expected that the cardiovascular and metabolic risk factors found in childhood and tracking into adulthood could be worse in later life, and may be responsible for chronic cardiometabolic disease^18^. This leads to the important question whether maternal metabolic information is passed on to offspring via epigenetic modification of the mitochondrial genome.

It has been discovered recently that like nuclear genomic DNA (gDNA), mtDNA can be methylated on cytosine residues^19^. Although not completely characterized, evidence is mounting that methylation plays a role in mtDNA gene expression and replication^20,21^. Furthermore, it appears that any cytosine nucleoside within mtDNA can be methylated, and is not restricted to CG dinucleotides^22^. This non-animal-cell characteristic^23^ also fits the recent discovery that oocytes bear substantially more non-CG methylation than somatic cells^24^. These new lines of evidence suggest that gene expression in oocytes and their mitochondria might be subject to a new context of DNA methylation either for an immediate or prolonged effect following fertilization and beyond. The aim of this study was to substantiate and explore the potential role of maternal mtDNA methylation in mitochondria on gene expression by comparing oocytes, blastocysts and granulosa cell mtDNA methylation using whole-genome bisulphite sequencing to determine (1) the effects of different ovarian environments on germinal vesicle oocyte (GVO) mtDNA methylation, (2) the developmental effects of these different ovarian environments on blastocyst mtDNA methylation, (3) the link between mtDNA methylation level and gene expression in oocytes and blastocysts, and (4) the effects of female maturity on granulosa cells mtDNA methylation. The results indicate that bovine mitochondrial DNA shows a unique signature in oocytes and blastocysts compared to the associated somatic (granulosa) cells; an inverted methylation-expression relation; and a specific pattern of mtDNA methylation associated with oocyte status/quality that may reflect programming of embryonic development potential.

## Results

The existence of mtDNA methylation is still controversial. This can be attributed to the unusual structure of mtDNA coupled with techniques used to study DNA methylation^25^. Here, we have optimized a method that includes a linearization step, enabling efficient mitochondrial WGBS^26^. Because of the size of mitochondrial DNA, we obtained significantly higher coverage of each cytosine (719 nucleoside sites) above 3,000-fold in oocytes samples compared to what is typically obtained for somatic cells (4-fold; only 38% of the cytosine were covered). This level of coverage provides extraordinary precision for mitochondrial DNA site-specific analysis and allows comparisons that could lead to better understanding of the precise role of DNA methylation in mitochondria. The purification approach used with granulosa cells was successful in enriching the contribution of mtDNA to the sequencing output by more than 100-fold, albeit with lower coverage to that obtained for oocytes and blastocysts.

With the capacity to accurately assess mtDNA methylation, we can now address many important questions about mtDNA methylation in reproductive biology. Thus, for this study, multiple samples were processed to substantiate and compare the effects of environmental exposures and maternal maturity on mtDNA methylation in oocytes, blastocysts and granulosa cells. For our experimental design, mitochondrial WGBS libraries from all samples were pooled in the same sequencing experiment using a multiplex linker, allowing statistical comparisons within and across samples.

**Figure 1:**
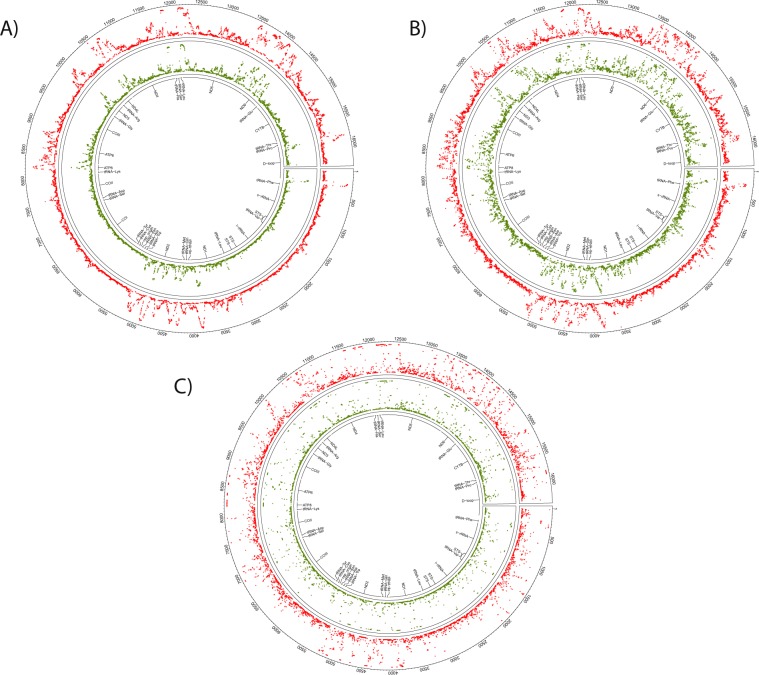
Circos representation of the total cytosine methylation intensities of the plus and minus strands of mitochondrial DNA in (**A**) oocytes, (**B**) blastocysts and (**C**) granulosa cells. The outer ring indicates the nucleotide position, while the inner ring shows the genes that encode proteins, tRNAs or rRNAs. (**A**) The red ring corresponds to DNA methylation in GVO from abattoir ovaries, while the green ring represents DNA methylation in OS/OPU GVOs. (**B**) The red ring depicts DNA methylation in blastocysts *in vitro* produced from GVOs from abattoir ovaries, while the green ring shows DNA methylation in blastocysts *in vitro* produced from OS/OPU GVOs. (**C**) The red ring corresponds to DNA methylation in granulosa cells from adult cows, while the green ring represents DNA methylation in granulosa from pre-pubertal cows.

**Figure 2:**
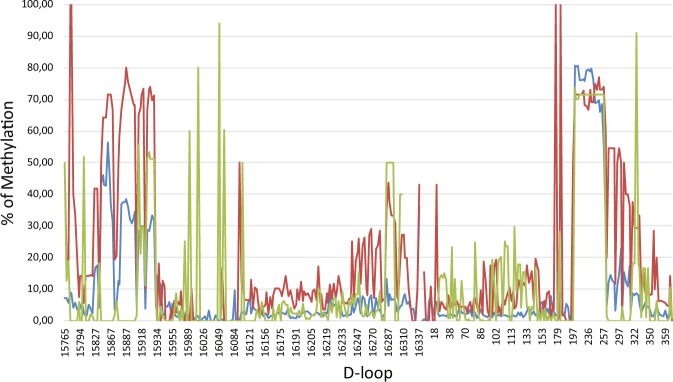
Representation of D-loop cytosine methylation intensity measured in OS/OPU GVOs (blue), blastocysts produced *in vitro* from OS/OPU GVOs (red) and granulosa cells from an adult cow (green).

Across sample comparison of overall mtDNA methylation between oocytes (Fig. 1A), blastocysts (Fig. 1B), and granulosa cells (Fig. 1C) revealed unique methylation patterns. However, the mtDNA methylation patterns were more highly conserved between oocytes and blastocysts compared to somatic (granulosa cells) (Fig. 1, Table 1). The similarities between oocytes and blastocysts was more apparent at higher magnification as shown for a representative region of mitochondrial DNA, the D-Loop (Fig. 2). Consistent with a previous report^22^, cytosine methylation occurred in multiple contexts, CG, CHG and CHH. As shown in the representative D-Loop region, CHH methylation is the most common compared to CG or CHG in all three sample types (Suppl. Fig. 1). Furthermore, positions of the CG, CHG and CHH methylation was more conserved between GVOs and blastocysts compared to granulosa cells.

**Table 1:** Across-group correlations of mtDNA methylation.

	Correlation	P value
GVO-OS/OPU vs BL-OPU	0.8139	<0.0001
GVO-OS/OPU vs BL-Abattoir	0.8587	<0.0001
GVO-OS/OPU vs GC-Adult	0.4291	<0.0001
GVO-OS/OPU vs GC-Prepub	0.1667	<0.0001
GVO-Abattoir vs BL- OS/OPU	0.8708	<0.0001
GVO-Abattoir vs BL-Abattoir	0.9089	<0.0001
GVO-Abattoir vs GC-Adult	0.4379	<0.0001
GVO-Abattoir vs GC-Prepub	0.1681	<0.0001
BL-OS/OPU vs GC-Adult	0.4599	<0.0001
BL-OS/OPU vs GC-Prepub	0.1502	<0.0001
BL-Abattoir vs GC-Adult	0.4495	<0.0001
BL-Abattoir vs GC-Prepub	0.1521	<0.0001

To validate the mitochondrial WGBS data, pyrosequencing was performed on selected sites and results for the D-Loop and ND4L regions of the mitochondrial genome are presented in Suppl. Fig. 2. Although the levels of methylation measured by this second approach on a different set of samples differ slightly from the mitochondrial WGBS results, they confirm that these sequences are methylated to similar degrees, and that they do not vary much from sample to sample, based on standard deviation (Suppl. Fig. 2).

**Figure 3:**
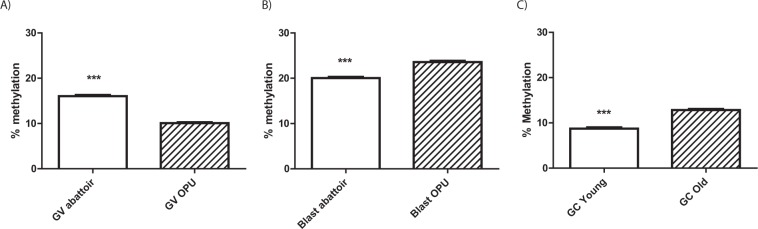
Comparison of total mtDNA methylation (both strands) in (**A**) GVOs collected from abattoir ovaries or obtained by OS/OPU, (**B**) blastocysts produced *in vitro* from abattoir or OS/OPU GVOs, and (**C**) granulosa cells obtained from FSH-stimulated adult or pre-pubertal cows.

To determine the developmental effects of these different ovarian environments on mtDNA methylation, blastocysts generated from abattoir oocytes were compared to those generated from high-quality OS/OPU oocytes. While the total cytosine methylation of mtDNA was highly correlated between the two groups of blastocysts (Table 1), a comparison of total cytosine methylation revealed a significantly lower level in blastocysts produced from GVOs recovered abattoir ovaries compared to blastocysts produced from OS/OPU GVOs (Fig. 3). Similar observations were found for CG, CHG and CHH methylation (Suppl. Fig. 3).

**Figure 4:**
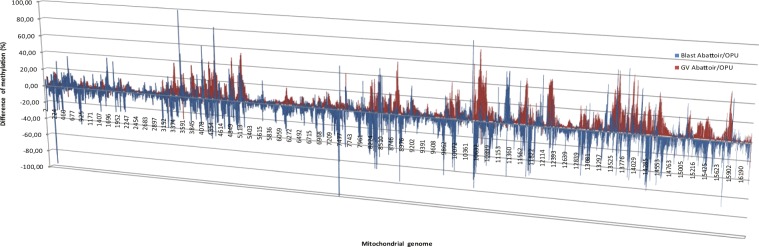
Site-by-site comparison of differences in mtDNA methylation (both complete strands) between GVOs (abattoir and OS/OPU) (red) and *in vitro* produced blastocysts (from abattoir and OS/OPU GVOs) (blue).

Comparing mtDNA methylation between GVOs and blastocysts with an abattoir origin verses those from OS/OPU produced a somewhat surprising finding. While total mitochondrial cytosine methylation was higher in oocytes recovered from abattoir ovaries than in OS/OPU oocytes, the opposite was the case in blastocysts obtained from these GVOs. A site-by-site comparison of differences in mtDNA methylation between GVOs (abattoir and OS/OPU) and *in vitro* produced blastocysts demonstrated these reciprocal patterns (Fig. 4).

Finally, since granulosa cells are linked to developmental competence of their associate oocytes^27^, we assessed the effects of maternal maturity on mtDNA methylation. Granulosa cells were obtained from hormone-stimulated, large (5–12 mm) follicles of pre-pubertal cows and adult cows. Total cytosine methylation was significantly lower in pre-pubertal heifers compared to adult cows (Fig. 3). This was true for all cytosine positions (Suppl. Fig. 3).

**Figure 5:**
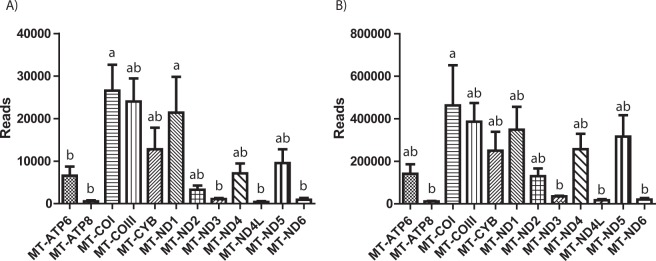
Representation of the number of reads for 12 mitochondrial transcripts obtained from GEO Series GSE52415 (31) in (**A**) oocytes and (**B**) blastocysts.

**Figure 6:**
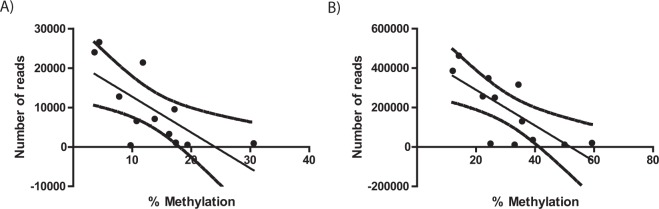
Analysis of correlation between the average number of reads for 12 mitochondrial transcripts obtained from GEO Series GSE52415 (31) and the average % methylation (total cytosines)(based on bisulphite) of the same bovine genes in (**A**) Oocytes, (**B**) Blastocysts.

**Figure 7:**
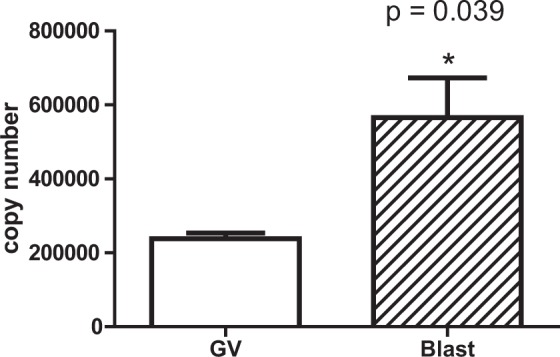
Abundance of mitochondrial DNA copies in GVOs and blastocysts. The mtDNA D-loop region was quantified by qPCR to obtain an estimation of the mitochondria DNA copy number present. The numbers were corrected to show mtDNA copy number for individual oocytes or blastocysts.

To determine whether there is a correlation between cytosine methylation and expression of genes in the mitochondrial genome, we investigated RNAseq data (GEO Series GSE52415) performed on oocytes and blastocysts for 12 mitochondrial-encoded genes using data from comparable stages to our experiment^28^. The level of expression obtained for the 12 genes in number of reads is depicted in Fig. 5. We first compared overall mitochondrial gene expression levels to total mtDNA cytosine methylation levels. A simple correlation analysis between the average methylation status of all cytosines in a given gene and the relative number of reads in the dataset revealed a strong negative correlation for both oocytes and blastocysts (Fig. 6). Next, we compared total mtDNA cytosine methylation levels at specific genes to their gene expression levels. Five genes (COX1, COX3, CYB, ND1, ND4L) possessed lower and 7 genes (ATP6, ATP8, ND2, ND3, ND4, ND5, ND6) had higher cytosine methylation levels in GVOs and blastocysts, generally with methylation levels higher and broader in the latter (Fig. 1). For both samples, the lower methylation levels for COX1, COX3, CYB and ND1 were associated with higher gene expression levels as determined by read count (Fig. 5). The reverse was seen for ATP6, ATP8, ND2, ND3, ND5, ND6, where higher methylation levels were associated with lower expression levels. Between sample comparison showed that the overall, gene expression levels were higher in blastocysts compared to oocytes. One explanation for higher transcript abundance could be higher numbers of mtDNA copies in blastocysts than in oocytes. To test this, we measured the mtDNA copy number in GVOs and blastocysts (abattoir). Here, mtDNA copy number was on average 3 times higher in blastocysts (Fig. 7).

## Discussion

This study is the first to compare mitochondrial DNA methylation in reproductive tissues. We found a more conserved mtDNA methylation signature between oocytes and blastocysts compared to the granulosa cells. This suggests that the oocyte methylation pattern may be inherited by preimplantation embryos. This seems reasonable since mitochondria in embryos are directly inherited from oocytes, with no substantial paternal contribution. Alternatively, CG methylation may be inherited from oocytes and act as nucleation centers for subsequent CHG and CHH methylation in preimplantation embryos. Furthermore, our data indicate different regulatory mechanism between germ/embryonic and somatic cell mtDNA methylation. Consistent with this, comparison of our methylation data with tissues in humans indicates that the observed mtDNA methylation signature associated with granulosa cells is more typical of the somatic environment (% methylation very high or very low at most sites) in comparison to the different patterns observed in oocytes and embryos^29^.

In addition to nuclear CG methylation observed in mammals, CHG and CHH methylation account for nearly two thirds of the methyl-cytosines present in mouse germinal vesicles and that these accumulate genome-wide in close proximity to methylated CG sites in growing oocytes^30^. The *de novo* DNA methyltransferase proteins, DNMT3A and DNMT3L, are likely responsible for non-CG methylation in oocytes. While it is difficult to imagine such proteins entering the mitochondria, it seems equally unlikely that only mitochondrial DNMT1 is responsible for non-CG methylation. Other gene products could be involved in non-replicative (symmetrical) distribution of methylation outside of CG. Of the three currently known DNA methyltransferases, only DNMT1 has been shown to target mitochondria. A recent paper describes a specific isoform of DNMT1 that might enter mitochondria^31^. Our analysis of RNA bovine sequences present on NCBI indicates a positive mitochondrial localisation sequence within some of the isoforms listed using IPSORT prediction algorithm (http://ipsort.hgc.jp/index.html). Observations in both this study and elsewhere (with mice) suggest that non-CG methylation occurs inside mitochondria and that oocytes would support a less specific catalytic action of such an isoform. In a recent study of melatonin-treated swine oocytes, DNMT1 expression was increased and the protein was trans-located into mitochondria and the inhibitory effect of melatonin on mtDNA expression was suppressed by simultaneous addition of a DNMT1 inhibitor, suggesting that melatonin regulates mtDNA transcription through up-regulation of DNMT1 and mtDNA methylation^32^. DNMT1 expression is also up-regulated by nuclear respiratory factor 1 (NRF1) and peroxisome proliferator-activated receptor gamma co-activator 1-alpha (PGC1α), transcription factors that activate expression of nuclear-encoded mitochondrial genes in response to hypoxia, and by loss of tumour suppressor protein 53 (TP53), known to regulate mitochondrial metabolism^33^. Overall, our investigation suggests a need to increase our understanding of the DNA methyltransferases involved in mitochondria CG as well CGH and CHH methylation in oocytes and preimplantation embryos.

In this study, we also compared the effects of different ovarian environmental exposures and maternal maturity on mtDNA methylation in oocytes, blastocysts and granulosa cells. We found significant changes in mtDNA methylation in oocytes and blastocysts based on the ovarian environment, which may indicate sensitivity to oocyte status/quality and subsequent mtDNA methylation programming of embryonic development. It is believed that the oocyte metabolism changes during oocyte development as the oocyte mitochondria have a different, immature structure, and are rather quiet compared to somatic tissues^34^. This shutting down of mitochondria may occurs progressively and could be associated with the general de-methylation of the mtDNA, which could explain why the abattoir oocytes (small follicles) had higher methylation levels than OS/OPU oocytes (large follicles). Generally, blastocysts possessed a higher level of mtDNA methylation with a broader distribution than oocytes, suggesting subsequent mtDNA methylation programming during embryonic development. However, these levels were reduced in blastocyst produced from abattoir oocytes than OS/OPU oocytes, suggesting that the developmental programming of mtDNA methylation was compromised in the less matured abattoir samples.

Many questions remain regarding mtDNA DNA methylation in oocytes and preimplantation embryos. Firstly, in addition of DNA methyltransferases and mtDNA methylation, a greater understanding is required for mechanisms of mtDNA demethylation during gametogenesis. The identification of TET proteins in mitochondria^35^ suggest that they could play a similar role as in the oocyte nuclear genome. Secondly, studies need to be done to analysis mtDNA methylation programming during oogenesis similar to those done for nuclear DNA. This will determine whether the gamete-specific pattern is established initially in early germ cells or is acquired as mitochondria adopt the oocyte-specific form during oocyte growth and maturation. Analysis during preimplantation development will also determine whether mtDNA methylation is inherited from the oocyte to the zygote, and subsequently expanded during preimplantation development. Analyses performed post-implantation will reveal when the demethylated somatic mtDNA methylation pattern, as observed in granulosa cells, is established, as well as whether early lineages or stem cells maintain the embryonic pattern or harbor a somatic program.

Many questions also arise regarding mtDNA DNA methylation and adverse environmental exposures. We observed that suboptimal ovarian environment and maternal maturity were associated with altered mtDNA methylation. Previous studies have revealed that the culture environment of preimplantation embryos not only induced up-regulation of mitochondrial RNA polymerase (POLRMT, a key regulator of mtDNA transcription and replication) at the blastocyst stage, but also increased mitochondrial ND4 mRNA preceding the blastocyst stage, suggesting that impaired regulation of mtDNA may be responsible for the abnormal metabolism and physiology and hence decreased viability of cultured embryos^36^. Further investigation of various culture environments and medium supplementation with metabolites such as folate will provide further insight into the regulation of mtDNA methylation.

Granulosa cells have been studied as a biomarker for oocyte developmental competence^37^. Our analysis revealed a difference in mtDNA methylation between the granulosa cells of adult and pre-pubertal cows. These patterns could reflect the linear process of age, or a follicle-specific pattern associated with the ovarian context or the metabolism of a maturing animal compared to an adult. To further explore this observation, follicles of different sizes within the same female should be compared for mtDNA methylation. In cows, the number of mitochondria doubles with age and also during oocyte maturation *in vitro*^38^ and exceeding a certain minimal number appears to be necessary for normal early development^39^. Furthermore, mitochondria methylation has been shown to increase with age in human and in several tissues that have been measured^20^. To assess linear aging, other tissues within prepubertal and adult females should be compared to assess if the ovary/follicle has different mtDNA methylation programming. Additional studies should examine mtDNA methylation in females of advanced maternal age.

An important question still revolves around the the potential function of the mtDNA methylation. A recent paper indicated that mitochondrial nuclear genes undergo *de novo* promoter methylation, which may have functional consequences on mitochondrial activity and dynamics during early development^21^. In this study, we examined mtDNA methylation levels and expression of mitochondrial genes. We observed that mtDNA methylation was negatively correlated with individual gene expression. More specifically, genes with higher methylation were associated with lower expression, while genes with low methylation levels had higher expression levels. We also found that gene expression levels were higher in blastocysts compared to oocytes. Higher transcript abundance in blastocysts could be explained by the greater mtDNA copy number in blastocysts (3 times), however, this does not explain the 20 times rise in transcript abundance. Instead, it is likely that the increase of transcription capacity comes from the nucleus in the form of polymerase and transcription factors. Three important transcription regulators, TFAM, Tfb2m and Polrmt, are transcriptional upregulated relative to other genes at the 8-cell stage in bovine embryos (http://emb-bioinfo.fsaa.ulaval.ca/IMAGE), perhaps additionally accounting for the robust expression in blastocysts. Further investigation of somatic tissues, such as liver, muscle and skin that have different mitochondria levels, could be used to study the role of mtDNA during transcription and replication, nuclear-mitochondrial co-regulation, as well as effects of mitochondrial DNA mutations.

In a broader context, the major role of mitochondria is to provide ATP, and their functionality is therefore closely related to metabolism. With respect to reproductive biology, mtDNA sequences encoding RNR1, RNR2 and ND4 as well as the D-loop region have been found significantly hypermethylated in porcine oocytes in association with PCOS, indicating that abnormal activation of one-carbon metabolism and hypermethylation of mtDNA may contribute substantially to mitochondrial malfunction and decreased oocyte quality^40^. In pigs, a low-protein diet during pregnancy was associated with sex-dependent epigenetic alterations in the expression of mtDNA-encoded oxidative phosphorylation genes and that the glucocorticoid receptor (NR3C1) is involved in mtDNA transcription regulation^41^. In humans, mtDNA methylation is associated with early insulin sensitivity and body mass index. An increase in BMI from lean to obese (20–24.9 to 30–34.9) coincides with a drastic increase in mtDNA methylation, while increases from obese to severely obese (35–39.9) and from severely to morbidly obese (BMI > 40) coincide with marginal increases in methylation^42^. Further investigations are required to determine the linkages between mtDNA methylation, mitochondrial function and cellular metabolism.

## Conclusion

The results presented here show that mtDNA methylation patterns in bovine oocytes and embryos differ from those in the associated granulosa cells and therefore warrant investigation of how such patterns control replication and transcription of mtDNA in both embryonic and somatic tissues. Secondly, the observed differences in oocyte quality and reflected in blastocyst methylation patterns may be indicative of late oogenesis/folliculogenesis programming, which likely has an impact on embryo quality or on the metabolic programming of the foetus or of the offspring.

## Materials and Methods

### Samples

Pools (n = 10) of germinal vesicle oocytes (GVO) were collected from 2–5 mm follicles from ovaries obtained from randomly selected animals at a local abattoir and also from females subjected to ovarian stimulation protocol (OS) followed by trans-vaginal ultrasound aspiration through a process called ovum pick-up (OPU)^43^ (5–12 mm follicles) in peri-pubertal animals (8–16 months old) obtained from a commercial IVF company (Boviteq, St Hyacinthe, Canada). The procedure allows for oocyte to remain in the growing follicle under FSH (Follicle stimulating hormone) stimulation which become dominant after a few days increasing the quality of the enclosed oocytes compared to oocyte collected from random non-stimulated animal at slaughter. Both types of oocytes are at the same maturation stage (GV) at collection but if exposed to similar culture conditions will lead to blastocysts rates of >50% for the stimulated compared to <30% for the abattoir derived^44^. Day-7 blastocysts were *in vitro* produced using *in vitro* maturation, fertilization and embryo development from abattoir GVO^45^ or OPU GVO^37^. Granulosa cells were obtained from hormone-stimulated 113-month-old (follicles 5–12 mm, adult) or 6-month-old (follicles 5–12 mm, prepubertal) cows by trans-vaginal ultrasound aspiration^44^. Experiments took place in compliance with the guidelines of the Canadian Council on Animal Care and supervised by the Animal Protection Committee of Université Laval. These guidelines are strictly followed by Boviteq, who provided all the tissues and samples. The study did not require handling of animals on university premises.

### Extraction of mtDNA from granulosa cells

The vast mitochondrial abundancy in oocytes and preimplantation embryos (>200,000 mtDNA copies) does not require mitochondrial enrichment prior to mtDNA purification. For granulosa cells, an enrichment procedure was use where mitochondria were isolated with hypotonic buffer followed by differential centrifugation^25^. Briefly, cells were suspended in 500 µL of RSB (1X:10 mM Tris-Cl, pH 7.4, 10 mM NaCl and 3 mM MgCl_2_) hypotonic buffer in a 1.0 mL glass homogenizer tube using 10 strokes in a B-pestle, diluted with 350 µL of 2.5X MS buffer, and then centrifuged at 1,300 × g for 5 min at 4 °C. The pellet was resuspended, washed with 500 µL of 1X MS buffer and spun again (1,300 × g for 5 min at 4 °C), following which the two supernatants were pooled in a clean tube and centrifuged at 16,000 × g for 15 min at 4 °C. The pellet was re-suspended in 500 µL of 1X MS buffer and washed (16,000 × g for 15 min at 4 °C). Mitochondrial DNA was extracted from the final pellet using ZR Genomic DNA – Tissue MicroPrep (Zymo Research) and then suspended in 40 µL of DNA elution buffer. MtDNA was digested with plasmid-safe ATP-dependent DNase (Epicenter) in a final volume of 50 µL for 2 h at 37 °C followed by 30 min at 70 °C, and then purified using ZR Genomic DNA – Tissue MicroPrep without proteinase K digestion. The purified DNA was suspended in 10 µL of DNA elution buffer.

### Whole-genome bisulphite sequencing (WGBS) library preparation

Pools of 10 GVO or 10 blastocysts were digested with proteinase K for 20 minutes at 50 °C in M-digestion buffer (1X) to obtain a final volume of 20 µL. Granulosa cell mtDNA was mixed with 10 µL of 2X M-digestion buffer and brought to a final volume of 20 µL using the reagents from the EZ DNA Methylation-Direct kit (Zymo Research). Since it has been reported that accessibility of the human mtDNA nucleoid to sodium bisulphite varies can compromise the mtDNA methylation analysis^26^, a restriction enzyme was used (200 U of Sal1 for 4 h at 37 °C) to linearize the bovine mtDNA samples. Next, samples were treatment with 130 µL of Lightning Conversion Reagent from the Pico Methyl-Seq Library Prep kit (Zymo Research). The WGBS library protocol was followed as recommended by the manufacturer for 10 pg to 1 ng of DNA with 10 cycles for the final two PCR amplification rounds. Purified libraries were suspended in 12 µL of DNA elution buffer.

### Library quantitation and sequencing

Six libraries prepared with the Pico Methyl-Seq Library Prep kit (Zymo Research) and the Illumina Index linker-primers for multiplexing (Illumina) were quantified, pooled and sequenced on one lane of Hiseq2500 SR 50 bp (Illumina). Library quantitation, quality control and sequencing protocol were performed at the McGill University and Génome Québec Innovation Centre (Montreal, Canada). The sequences were analysed against the reference bovine mitochondrial bisulphite-treated DNA, and the ratio of methylation was analysed for each individual site, and for the 3 types of cytosine positions (CG, CHG, CHH). A Fisher-type analysis was performed with false discovery rate (FDR) filtering. The data discussed in this paper have been deposited in the National Center for Biotechnology Information (NCBI) Gene Expression Omnibus (GEO) and are accessible through GEO Series Accession number GSE122599.

### Sequences bioinformatics analysis

Using Trimmomatic, sequencing adapters were removed and base calls with a quality score below 30 were removed from the ends of the reads^46^. Reads with a minimal length of 50 nucleotides were kept for further processing. Methylation calls for the bovine mitochondrial genome (NC_006853.1) were then obtained using bismark^47^.

### Pyro-sequencing

To validate WGBS cytosine methylation results, we performed pyro-sequencing for several methylation sites in the D-Loop and ND4L regions of the mitochondrial genome. Individual blastocysts (n = 6) produced *in vitro* as described previously were treated with sodium bisulphite directly using the EZ DNA Methylation-Direct kit. PCR was performed for 2 regions on mtDNA, namely the D-loop and ND4L, in 25 µL using the PyroMark PCR kit (Qiagen) in 1X buffer, 3 mM MgCl_2_ and 5 µL bisulphite-modified DNA (corresponding to 0.1 blastocyst). Primers information are presented in Suppl Table 1). The cycling conditions were as follows: 95 °C for 15 min followed by 45 cycles of 30 sec at 95 °C; 30 sec at 52 °C; 30 sec at 72 °C; and a final elongation step of 10 min at 72 °C. PCR products were verified by electrophoresis on 2% w/v agarose gel and then attached to streptavidin-coated Sepharose beads (GE Healthcare) in the presence of binding buffer (Qiagen). The pyro-sequencing reactions were performed on a PyroMark Q24 Advanced system using the PyroMark Q24 advanced CPG reagents kit (Qiagen) in accordance with the manufacturer’s recommendations. The pyrosequencing assay design was done using the Pyromark assay design 2.0 (Qiagen).

### Mitochondrial DNA copy number quantification

Pools of 20 GVO (n = 3) collected from abattoir and 7-day blastocysts (n = 3) produced *in vitro* from these GVOs were extracted using ZR Genomic DNA – Tissue MicroPrep for gDNA and mtDNA. Primers for the D-loop region on mtDNA (Suppl Table 1) were designed using the IDT PrimerQuest tool (http://www.idtdna.com/Scitools/Applications/Primerquest/) from sequences obtained using the Genebank V00654.1 *Bos taurus* complete mitochondrial DNA. To confirm primer specificity, the amplified fragment was purified by electrophoresis on a standard 1.2% agarose gel then quantified and sequenced. The products were then used to create the standard curve for quantification, with dilutions ranging from 2 × 10^−4^ to 2 × 10^−8^ ng/µL. Realtime PCR was performed on a LightCycler 480 (Roche Diagnostics) using LightCycler 480 SYBR Green I Master. Each qPCR reaction mixture (final volume of 20 µL) contained the mtDNA from single GVOs or blastocysts. The PCR conditions were as follows: denaturing cycle for 10 min at 95 °C; 50 PCR cycles (denaturing at 95 °C for 5 sec; annealing at 57 °C for 5 sec; extension at 72 °C for 5 sec; melting curve (94 °C for 5 sec, 72 °C for 30 sec; and ramping up to 94 °C at 0.2 °C/sec); and a final cooling step at 40 °C. Quantification was performed with LightCycler 480 Software version 1.5 (Roche Diagnostics) by comparison to the standard curve. PCR specificity was confirmed by melting-curve analysis. Mitochondrial DNA copy numbers were calculated from the standard curve.

### Mitochondrial RNA analysis

For the level of expression of mitochondrial genes, 12 mitochondrial transcripts abundance were obtained from GEO Series GSE52415 acquired from sequencing pools of 10 denuded bovine oocytes and 10 blastocysts produced *in vitro*^28^. Briefly, the libraries were prepared with Ovation RNAseq v2 kit (NuGEN) and the barcoded libraries were pooled for multiplexed sequencing on an Illumina GAIIx to a mean coverage of 20 × 10^6^ reads each. Sequencing runs were done in single-read mode with an 80-base read length.

### Statistical analyses

All statistical analyses were performed using Graphpad prism 5 software. An unpaired t-test analysis was performed for total mtDNA methylation, mtDNA methylation at specific cytosines, and mtDNA abundance. Anova analysis was performed for the mRNA gene-specific expression analysis and a Pearson r correlation analysis for the methylation/reads analysis.

## Supplementary information

Supplemental material
