# Supplementary material for: Distribution and dynamics of mitochondrial DNA methylation in oocytes, embryos and granulosa cells
Source: Sci Rep. 2019 Aug 15;9:11937. doi: 10.1038/s41598-019-48422-8 (PMC6695495; doi:10.1038/s41598-019-48422-8)
Supplement: Supplementary file 1 — Supplemental material [file 41598_2019_48422_MOESM1_ESM.pdf]

Supplemental material

Title: Distribution and dynamics of mitochondrial DNA methylation in oocytes, embryos and granulosa cells

Authors and Affiliations:

Marc-André Sirard

Centre de recherche en reproduction, développement et santé intergénérationnelle (CRDSI)

Département des Sciences Animales, Faculté des sciences de l'agriculture et de l'alimentation, Université Laval, Québec, Canada

Correspondence:

Marc-André Sirard, Centre de Recherche en Biologie de la Reproduction, Département des Sciences Animales, Université Laval, Québec (Québec) Canada G1V 0A6

E-mail: Marc-Andre.Sirard@fsaa.ulaval.ca

Phone: 418-656-2131 ext. 7359; Fax: 418-656-3766

Suppl. Table 1: Primers used for the pyro-sequencing reactions and mtDNA quantification

		Primers	Cytosine
D-Loop	F	5’-GTTTTGTAAATTAGAGAAGGAGAATAA-3’	CHH-15886
(pyro-sequencing)	R	5’-Biotin-ACATTAATATTATATACATTACCCCTT-3’	CHH-15887
	S	5’-GGATAATTTAATATAGAATTTGTATTT-3’	CHH-15913
			CG-15920
MT-ND4L	F	5’-GTGGATTTGATTTTATAGGATTAGT-3’	CHH-10033
(pyro-sequencing)	R	5’-Biotin-TTCTAATCCTTTTTAAATTCACTCATA-3’	CHH-10036
	S	5’-ATTTTTTTTATTTGATTTAGAAATT-3’	CHH-10060
			CHG-10064
D-Loop	F	5’-GACATCTCGATGGACTAATGGCTAATCAG-3’	
	R	5’-CCTGTAACCATTGACTGTAATGTCC-3’	

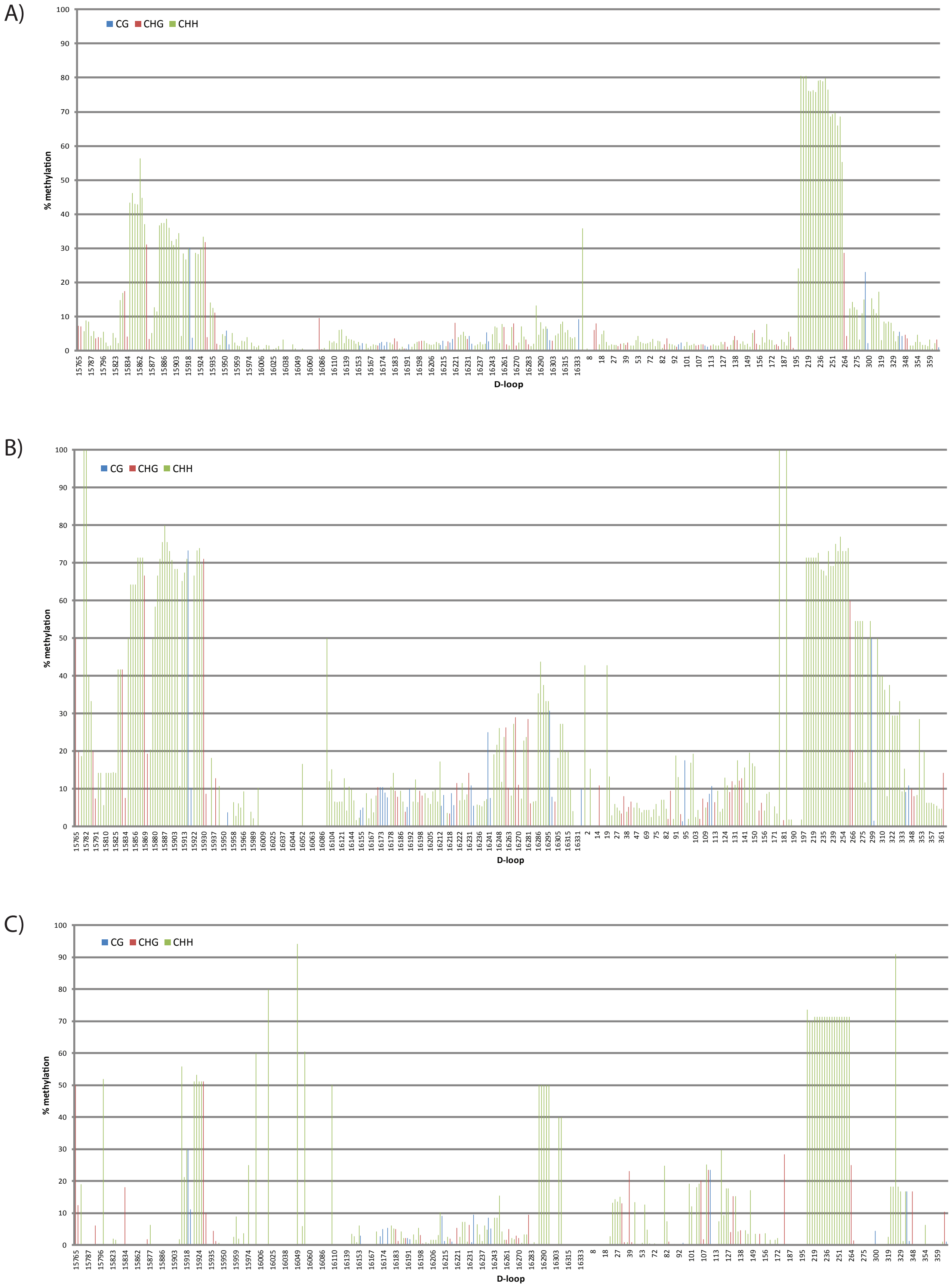

Suppl fig 1 Distribution of cytosine methylation (CG in blue, CHG in red, CHH in green) in the mtDNA D-loop region (both strands) in (A) GVO (OS/OPU) , (B) blastocysts (OS/OPU), and (C) adult granulosa cells.

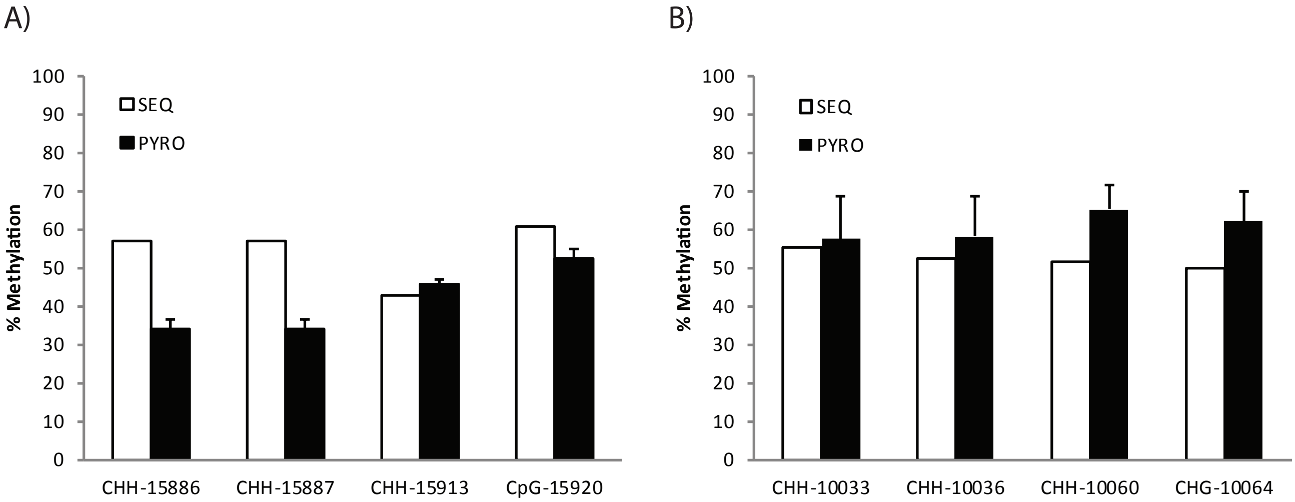

Suppl fig 2 . Cytosine methylation at specific positions (CG, CHG or CHH type indicated) in bovine mtDNA as measured using bisulphite (white) and pyro-sequencing (black), the latter plotted with standard deviation (n = 6 individual blastocysts), A) D-Loop region, B) ND4 gene region.

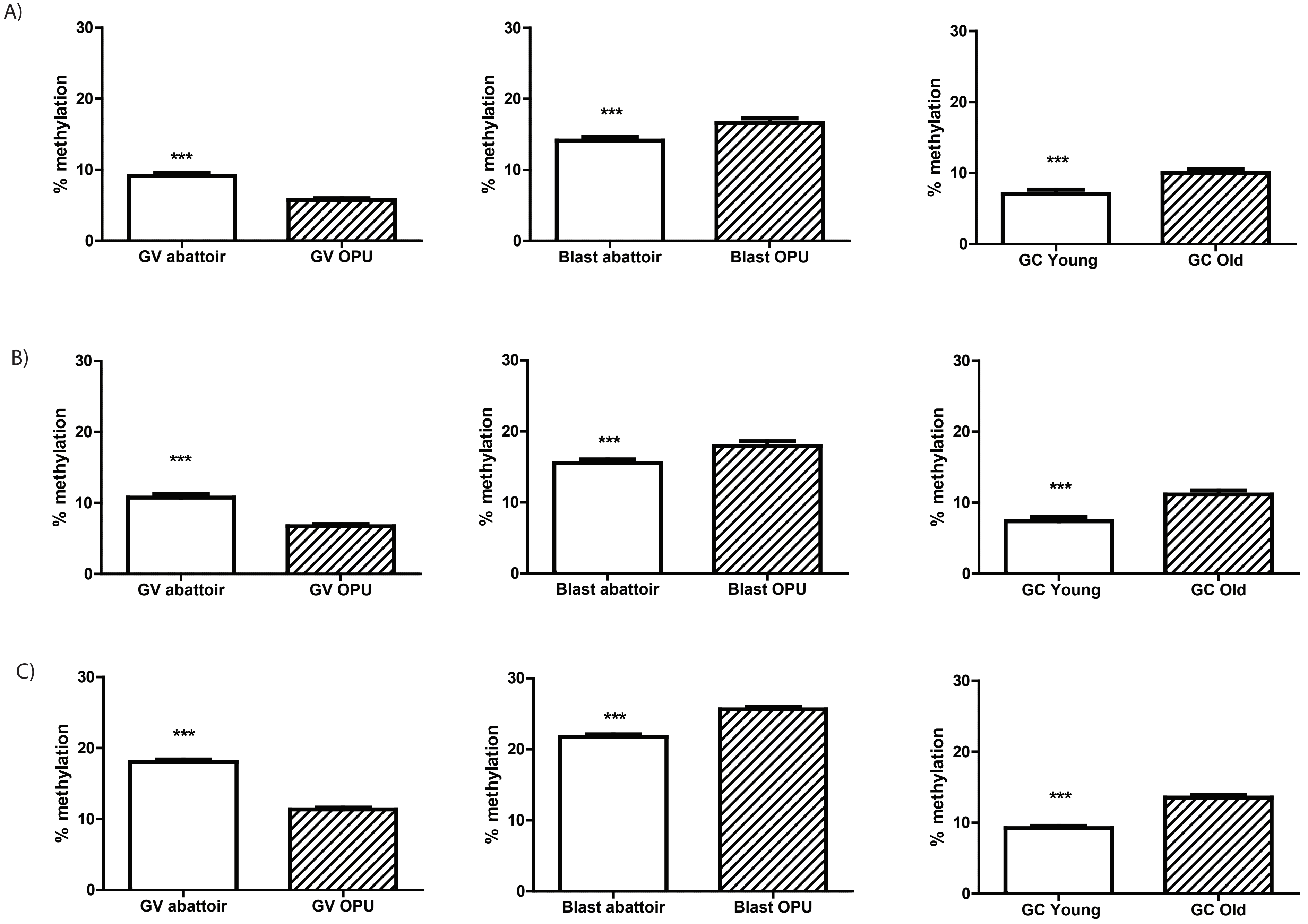

Suppl fig 3. (A) CG, (B) CHG, and C) CHH methylation of mtDNA (both strands) in GVOs recovered from abattoir ovaries or obtained by OS/ OPU (left), in blastocysts produced from these GVOs (middle), and in granulosa cells from adult or prepubertal cows (right).
